# Chemical profile of *Ficus lyrata* bark extract and its therapeutic effect on non-alcoholic fatty liver disease via regulating oxidative stress, inflammation and hepatic lipogenesis

**DOI:** 10.1186/s12906-025-05010-w

**Published:** 2025-07-19

**Authors:** Amria Mamdouh Mousa, Rehab Fikry Taher, Nermin Mohamed El-Sammad, Esraa Aly Balabel, Elham Mohamed Youssef, Ahmed Hassan Afifi, Sahar Samir Abdel-Rahman, Nayera Anwar, Sherien Kamal Hassan

**Affiliations:** 1 Biochemistry Department, National Research Centre, Giza, Egypt; 2 Chemistry of Natural Compounds Department, National Research Centre, Giza, Egypt; 3 Cell Biology Department, National Research Centre, Giza, Egypt; 4 Pharmacognosy Department, National Research Centre, Giza, Egypt; 5 Pathology Department, Faculty of Veterinary Medicine, Cairo University, Cairo, Egypt; 6 Pathology Department, National Cancer Institute, Cairo University, Cairo, Egypt

**Keywords:** Anti-inflammatory, Antioxidant, *Ficus lyrata*, High fat diet, Non-alcoholic fatty liver disease, Polyphenols

## Abstract

The high prevalence of non-alcoholic fatty liver disease (NAFLD) worldwide necessitates the attention and intervention of modern medical treatment options. Preliminary studies have demonstrated that *Ficus Lyrata* leaves can exert protective effects in rats against hepatic fibrosis and hypercholesterolemia. Hence, this study was conducted to investigate the therapeutic effect of *Ficus lyrata* Wrab bark extract on the NAFLD rat model. NAFLD was induced through a high-fat diet (HFD) for 12 weeks in male Wistar rats. After four weeks of HFD feeding, the rats were treated with *F. lyrata* extract (250 mg/kg, 5 days/week) or simvastatin (4 mg/kg, 5 days/week) while continuing on the HFD till the end of the experiment. Serum and liver samples were harvested for biochemical, molecular, histopathological, and immunohistochemical investigations. In silico analysis was also conducted to analyse the binding affinity of the extract polyphenols and the key regulators of hepatic lipogenesis. The results revealed that *F. lyrata* extract administered to HFD-fed rats significantly improved the characteristics of NAFLD by reducing hyperglycemia, hyperinsulinemia, and aminotransferases while improving serum lipid profile and adipokines. Moreover, the extract reduced the expression of hepatic lipogenic genes sterol regulatory element-binding protein-1c (SREBP-1c), acetyl-CoA carboxylase-1 (ACC-1), and fatty acid synthase (FAS), and inflammatory markers tumour necrosis factor-α (TNF-α) and interleukin-6 (IL-6), along with modulating oxidative stress markers and reversing histopathological changes. Molecular docking study revealed that most polyphenolic compounds in *F. lyrata* extract exhibited good binding affinity towards peroxisome proliferator-activated receptors γ (PPAR-γ) and liver X receptor α (LXR-α). In conclusion, our findings suggested that *F. lyrata* bark could be a promising therapeutic agent against the health issues related to NAFLD.

## Introduction

Obesity is one of the most common global metabolic diseases and is characterized by an imbalance between energy intake and expenditure. Recently, an observable increase in obesity has been detected with excessive consumption of high-fat foods, a rise in people’s living standards, as well as a westernized lifestyle [1]. Obesity is often considered a major risk factor for a variety of chronic metabolic diseases such as hypertension, type II diabetes, different types of cancer, and liver diseases [2]. Clinical and epidemiological studies have revealed that NAFLD often goes hand in hand with obesity [3].

NAFLD is one of the most extensive liver diseases worldwide and affects more than 25% of the world’s population [4]. It covers a wide variety of liver diseases, including simple steatosis, non-alcoholic steatohepatitis, fibrosis, and cirrhosis [5]. NAFLD is a multifactorial disease generally associated with oxidative stress, inflammation, and insulin resistance [6]. However, hypolipidemic drugs, antioxidants, and insulin sensitizers, the main drugs treating NAFLD, are accompanied by several side effects [7]. Currently, there is no effective pharmacological therapy for NAFLD. Therefore, the need is arising to develop new and safe strategies for NAFLD treatment.

Presently, the use of natural products as alternative medicines for treating many chronic diseases has increased rapidly all over the world. Thus, herbal medicines with lipid-lowering, anti-inflammatory, and antioxidant properties may be useful for NAFLD management, particularly phytochemicals such as flavonoids and phenols, which have been reported to modulate NAFLD progression [8, 9].

Ficus is a well-known genus in the Moraceae family enriched with a wide range of beneficial polyphenolic phytochemicals [10]. Ficus species are valuable therapeutic plants, well known for their actions against most oxidative stress illnesses and considered a potential candidate for hepatoprotection [11, 12]. *Ficus spragueana* Mildb‎r. & Burret produced pronounced suppression for the pro-inflammatory cytokines and enhanced hepatic regeneration and antioxidant defense system in the intrahepatic cholestasis rat model [12]. *Ficus religiosa* Linn. was reported to have significant hepatoprotective properties against hepatotoxicity in rats through its antioxidative effects [13]. Solanki et al. [14] found that *Ficus racemosa* bark extract has positive effects in the reversal of NAFLD in rats via a combination of antioxidant, hypolipidemic, and antidiabetic actions. *Ficus hirta* Vahl. roots exerted beneficial preventive and therapeutic actions on NAFLD by improving lipid metabolism and inflammation in a mouse model fed HFD [15]. *Ficus lyrata*, commonly referred to as the “fiddle-leaf fig, is a flowering plant native to Western Africa. It possesses promising pharmaceutical properties with potent antioxidant power and remarkable antimicrobial, antidiabetic, antifibrotic, and neuroprotective properties [16–19]. *F. lyrata* leaf extract was reported to possess notable hepatoprotective, hypolipedimic, and antioxidant effects in HFD-induced rats [18], which are the main therapeutic targets for NAFLD treatment. Awad et al. [18] declared that, diminished fat accumulation in the hepatocytes, and improvement in liver function, total lipid profile, and oxidative stress markers obtained by *F. lyrata* could be due to flavonoids and phenolics identified in the plant extract. Further, *F. lyrata* was confirmed to have a hypoglycemic effect [19], whereas hypoglycemic agents were reported to have positive effects on NAFLD treatment [20, 21]. Recently, Elganzoury et al. [19] stated that *F. lyrata* has the ability to mitigate inflammation and reverse the oxidative damage in vivo via ameliorating inflammatory markers and antioxidant indices.

As far as we know, there is currently no scientific data in the literature that recognises the chemical profile or proves the efficacy of *F. lyrata* bark extract in managing NAFLD. Therefore, the current study was designed to identify chemical compounds in *F. lyrata* bark extract and evaluate its efficacy in attenuating HFD-induced NAFLD in rats. The possible mechanisms that explain its protective effect were illustrated in terms of oxidative, inflammatory, hypolipidemic, hypoglycemic, and lipogenic machineries. Besides, molecular docking simulation was used to gain deeper insight into the lipogenic mechanism adopted by the phytochemical compounds presently identified in the extract.

## Materials and methods

### Plant collection and Preparation of *F. lyrata* bark extract

Fresh *F. lyrata* bark samples were collected from National Research Center Garden (June 2021). Plant authentication was performed by Dr. Mohamed El-Gebali, Botany Department, NRC, and deposited in the herbarium of National Research Centre (CAIRC) by Prof. Dr. Mona M. Marzouk with voucher number: M175. The powder of air-dried *F. lyrata* barks (3 kg) was extracted with CH_3_OH: H_2_O [7:3 (6 × 4 L)] at room temperature. All extracts were filtered and evaporated under reduced pressure to obtain a dry extract (300 g), to be redissolved in water for further chemical and biological investigations.

### Identification of phytochemical compounds in *F. lyrata* bark extract by HPLC-ESI-MS/MS

Sample solution (100 µg/ml) was prepared using high performance liquid chromatography (HPLC) analytical-grade MeOH, filtered using a membrane disc filter (0.2 μm), and then subjected to HPLC–ESI–MS analysis. Samples (10 µl) were injected into an HPLC instrument equipped with a reverse-phase C18 column (Acquity UPLC–BEHC18 1.7 μm particle size, 2.1 × 50 mm). Mobile phase elution was made with a flow rate of 0.2 ml/min using two eluents: eluent A is H2O acidified with 0.1% formic acid, and eluent B is MeOH acidified with 0.1% formic acid. The parameters for analysis were carried out using negative ion mode as follows: source temperature, 150 °C; cone voltage, 30 eV; capillary voltage, 3 kV; desolvation temperature, 440 °C; cone gas flow, 50 L/h; and desolvation gas flow, 900 L/h. Mass spectra were detected in the electrospray ionization (ESI) between m/z 100 and 1000. The peaks and spectra were processed using Maslynx 4.1 software and tentatively identified by comparing their retention time and mass spectrum with reported data. Precursor ions were selected and fragmented in the collision cell by applying collision energies in the range of 10–30 eV. Argon was used as collision gas. Product ions were detected using the following settings: pulse frequency, 10 kHz; spectral rate, 1.5 Hz. The collision-induced dissociation of the in-source fragment ions increased from 0 to 100 V. MS/MS spectra were obtained on a Thermo Orbitrap Fusion instrument using the same elution gradient as that in HPLC–MS. The system was equipped with an ESI source (electrospray voltage 4.0 kV, sheath gas: nitrogen; capillary temperature: 275 °C) in negative ionization modes. A group of authentic samples was used to compare the sample’s compounds for confirmation. Such authentics used were kaempferol glucoside, quercetin glucoside, Kaempferol, Quercetin, Isorhamnetin, Quinic acid, and p-Coumaric acid.

### Antioxidant activity of *F. lyrata* bark extract in vitro

The antioxidant activity of the *F. lyrata* extract was determined in terms of radical-scavenging ability using 2,2-Diphenyl − 1- picrylhydrazyl (DPPH) radical scavenging assay, as described by Ereifej et al. [22]. In brief, serial dilutions of trolox (4–20 µg/ml) or plant extract (10–50 µg/ml) in methanol were mixed with 3 ml of DPPH solution (50 mg/L). The mixture was then allowed to stand in dark for 30 min. The scavenging effect of DPPH was followed by monitoring the decrease in the absorbance (A) at 517 nm against methanol as a blank (control). The percentage of the radical scavenging activity (RSA) was calculated based on the following equation:

$${\rm{RSA }}\left( {\rm{\% }} \right) = {{\left( {{\rm{Absorbance}}\>\left( {{\rm{Control}}} \right) - {\rm{Absorbance}}\>\left( {{\rm{Sample}}} \right)\>} \right)} \over {{\rm{Absorbance}}\>\left( {{\rm{Control}}} \right)}} \times 100$$

The efficient concentration in µg/ml required to decrease DPPH radical concentration by 50% (IC_50_) was calculated graphically from linear regression analysis.

The antioxidant activity of *F. lyrata* extract was also reported as Trolox equivalents (TE)/g extract. It was quantified by reference to a Trolox standard calibration curve prepared using different concentrations (4–20 µg/ml).

### Subacute toxicity study

The subacute toxicity study was carried out on Swiss albino mice according to the OECD guidelines (Test No. 407) [23] for testing *F. lyrata* bark extract. Twenty male mice weighing between 20 and 25 g were chosen for the experimental trial. Mice were subdivided into four groups of five mice each. Three groups of mice were orally administered *F. lyrata* bark extract once daily at doses of 1000, 2500, and 5000 mg/kg body weight for 14 days. Mice were observed daily for toxic symptoms, mortality, or any abnormal findings.

### Experimental NAFLD model

#### Diet Preparation

The standard chow diet, used as control condition, was composed of 65 g carbohydrates, 20.3 g protein, 5 g fat, 5 g fibers, 3.7 g vitamins and minerals, and 1 g salt mixture per 100 gram diet; with energy sources: 67.3% by carbohydrates, 21% by protein and 11.7% by fat. For the HFD, we used 79% standard chow diet, supplemented with 20% melted sheep tallow and 1% cholesterol; composed of energy sources: 41.6% by carbohydrates, 13% by protein and 45.4% by fat. The macronutrient composition of the standard chow diet and the high-fat diet is summarized in Table 1.

**Table 1:** Macronutrient composition of the normal diet and high-fat diet fed to rats

	Experimental diet	
	Standard chow diet	High-fat diet
Carbohydrate % kcal	67.3	41.6
Fat % kcal	11.7	45.4
Protein % kcal	21	13
Energy (kcal/g)	3.86	4.94

#### Dosing Preparation

To prepare plant solution ready for dosing, we started by dissolving 10 g of dried *F. lyrata* bark extract in 100 mL of pure water to make a 100 mg/mL stock solution. Based on average animal weight, we calculated the suitable volume of the extract solution to achieve the 250 mg/kg dose.

In order to compare the results of *F. lyrata* extract, simvastatin (SMV) was selected as a lipid-lowering standard drug with a widespread clinical use and well-established efficacy in the management of hyperlipidemia and ameliorating hepatic fatty infiltration. The experimental effective dose (4 mg/kg) of SMV was chosen, where SMV was dissolved in double distilled water forming a solution at a concentration of 0.4 mg /ml, according to Gao et al. [24]

### Animals

Thirty male Wistar rats, (140 ± 10 g), were used for the HFD-induced NAFLD model. All Animals were obtained from the animal house of the NRC. They were housed in cages at 22 ± 2 °C, under a 12:12 h light/dark cycle, with a relative humidity of 50 ± 5%, and with free access to food and water. All experimental procedures were approved by the Medical Research Ethics Committee of the National Research Centre, Egypt with approval No: 23,152,032,021. The study is reported in accordance with ARRIVE guidelines.

### Experimental design

Rats were randomly divided equally into five groups on the basis of their body weights;

- Control group: Fed a standard chow diet.
- *F. lyrata* group: Fed a standard chow diet and received (250 mg/kg) *F. lyrata* bark extract.
- HFD group: Fed HFD for 12 weeks to establish the NAFLD model.
- HFD + SMV group: Fed HFD and treated orally with (4 mg/ kg) SMV solution.
- HFD + *F. lyrata* group: Fed HFD and treated orally with (250 mg/kg) *F. lyrata* bark extract.

The whole study lasted for 12 weeks with 8-weeks oral administration of simvastatin or *F. Lyrata* extract 5 days/week. The rats were biweekly monitored for the body weight The dose of *F. Lyrata* extract or SMV was changed according to body weight change. At the end of the experiment, body weight and naso-anal length (NAL) of the rats were measured to calculate the following anthropometrical parameters:

$${\rm{Body}}\>{\rm{mass}}\>{\rm{index}}\>\left( {{\rm{BMI}}} \right) = {{{\rm{body}}\>{\rm{weight}}\>\left( {\rm{g}} \right)} \over {{\rm{NA}}{{\rm{L}}^2}\>{{\left( {{\rm{cm}}} \right)}^2}}}$$

$${\rm{Lee}}\>{\rm{index}} = {{\root 3 \of {{\rm{body}}\>{\rm{weight}}\>\left( {\rm{g}} \right)} } \over {{\rm{NAL}}\>\left( {{\rm{cm}}} \right)}}$$

### Sample collection

At the end of the experiment (24 h after the last dose), i.p. ketamine-xylazine combination (50 mg/kg + 5 mg/kg) was used to anaesthetize 12 h fasted rats. This mixture was freshly prepared prior usage as follows: 1 mL of ketamine hydrochloride solution + 1 mL of xylazine solution. Blood samples were collected using heparinized capillary tubes from the retro-orbital venous plexus. The serum was separated by centrifugation and stored at − 80 °C to be used for biochemical assays. After confirming the animal death, the livers of rats were immediately removed, washed with ice-cold saline and weighed. A portion of the liver was used to prepare 10% liver homogenate. Another portion of liver tissue was snap-frozen at -80 ºC for gene expression analysis. The remaining portion of the livers was preserved in 10% formalin for histopathological and immunohistochemical investigations.

### Biochemical analysis

#### Serum biochemical parameters

Fasting blood glucose, total cholesterol (TC), high-density lipoprotein (HDL), triglyceride (TG), alanine aminotransferase (ALT), and aspartate aminotransferase (AST) were estimated by enzymatic colorimetric methods using commercial kits (Spectrum Diagnostics, Egypt) according to the manufacturer’s instructions. Very low-density lipoprotein (VLDL) and low-density lipoprotein (LDL) levels were calculated using a standard formula. Serum levels of insulin, leptin, and adiponectin were measured by ELISA kits supplied by Sunlong Biotech Co., Ltd. (China). The homeostasis model assessment of insulin resistance (HOMA-IR) index was calculated according to Matthews et al. [25] using the following equation:

HOMA-IR (µIU/ ml)/(mg/dl) = (Fasting serum insulin x Fasting serum glucose)/405.

#### Oxidative stress biomarkers

Hepatic activities of glutathione peroxidase (GSH-Px) and catalase (CAT) were determined according to the methods of Necheles et al. [26] and Aebi [27], respectively. Reduced glutathione (GSH) in the liver was investigated according to Beutler [28]. Hepatic malondialdehyde (MDA), a lipid peroxidation marker, was estimated according to Lefevre et al. [29] method. Total antioxidant capacity (TAC) was measured using a commercial kit (Biodiagnostic Research Reagents, Egypt).

### Gene expression analysis

#### Total RNA isolation and purification

GENEzolTM reagent was used to isolate total RNA from liver tissues according to the manufacturer’s protocol. RNA was resuspended in nuclease-free water and stored immediately at -80 °C. The concentration of harvested RNA was measured using a Nanodrp 1000 spectrophotometer (Thermo Scientific) and 1 µg of total RNA was cleaned from contaminated DNA using DNase I (Thermo Scientific).

#### Reverse transcription reaction and qRT-PCR

The first strand of cDNA was synthesized from purified RNA by RevertAid first strand cDNA synthesis kit (Thermo Scientific). The expression of three lipogenic genes; sterol regulatory element-binding protein-1c (SREBP-1c), acetyl-CoA carboxylase-1 (ACC-1), and fatty acid synthase (FAS), was evaluated in liver tissue using qRT-PCR, primer sequences are shown in Table 2. β-actin was used as an internal control. Reactions were performed in a Rotor GeneQ Qiagen PCR cycler using Enzynomics ToPrealTM qPCR 2X premix SYBR Green with low ROX, and the thermal profile was started at 95 °C for 15 min. followed by 40 cycles of 95 °C for 10 s, 60 °C for 15 s, and 72 °C for 30 s, and then finished with melting curve analysis ranging from 70 °C to 95 °C to evaluate the quality of the reactions. The threshold cycle (Ct) values of target genes were normalized to the Ct value of β-actin, and their relative quantification was calculated using the 2^-ΔΔCt^ method, as described by Livak and Schmittgen [30].

**Table 2:** The primers sequence of reference and target genes

Gene	Forward primer	Reverse primer
SREBP-1c	5`AGGAGGCCATCTTGTTGCTT3`	5`GTTTTGACCCTTAGGGCAGC3`
ACC-1	5` CCCTACACTTACTGATGAGC 3`	5` CTCTACTTGGTTATGGCGAA3`
FAS	5′ GAAGCATATCCCTGGAAACA 3′	5′ GACTCTTCTGGACACTCAAG 3`
β-actin	5′ CGTGCGTGACATTAAAGAGAA 3′	5′ CGCTCATTGCCGATAGTGAT 3′

### Histopathological investigation

Specimens of liver tissue fixed in 10% buffered neutral formalin were routinely processed for obtaining paraffin block sections of 5 μm and staining with hematoxylin and eosin (H&E). The histological lesions were described according to Brunt et al. [31], based on the percentage of involved hepatocytes and their zonal distribution, where; macrovesicular steatosis was quantified as follows: 0 absent; 1 (mild) less than 33% involvement of the hepatocytes; 2 (moderate)33–66% involvement of hepatocytes; 3 (severe) more than 66% involvement of the hepatocytes. Brunt et al. [31] methodology was used to determine the NAFLD activity score in which steatosis, lobular inflammation, and ballooning of hepatocytes were assessed.

### Immunohistochemical evaluation

A paraffin section from each group was used for immunohistochemical detection of the expression of tumor necrosis factor-α (TNF-α) and interleukin-6 (IL-6) using avidin-biotin peroxidase according to Haines et al. [32]. Liver sections were incubated with monoclonal antibodies against TNF-α and IL-6 at dilutions of 1:100 and 1:200, respectively (Abcam, Cambridge, USA) and avidin-biotin peroxidase (Vactastain ABC peroxidase kit, Vector Laboratories). The expression of each marker was visualized using the chromagen 3, 3-diaminobenzidine tetrahydrochloride (DAB, Sigma Chemical Co.). The positive brown area of each marker expression was quantified using image analysis software (Image J, 1.46a, NIH, USA) as the optical density in six high-power microscopic fields.

### Molecular Docking simulation

The identified polyphenols of *F. lyrata* bark extract were subjected to molecular docking studies. The docking study was performed using the open-source software Auto Dock Vina v1.1.2, with MGL tools v1.5.7 [33]. The X-ray co-crystal structures of PPAR-γ bound to the pioglitazone complex (PDB code: 5Y2O) and LXR-α bound to the GW3965 complex (PDB code: 3IPQ) were retrieved from the Protein Data Bank. The receptors were prepared by the addition of polar hydrogens, deletion of water molecules and other heteroatoms, and addition of Kollman Charges. The prepared receptors were saved in PDBQT format for molecular docking. All ligands were downloaded from the PubChem database, energy minimized with the force field MMFF94, and saved in the PDBQT format. The grid boxes were centered at the co-crystallized ligand for each receptor with dimensions of 30 × 30 × 30 Å and a grid spacing of 0.375 Å to include the entire binding site. Docking calculations were performed using Lamarckian Genetic Algorithm. The generated docking poses were ranked according to their docking scores, and the best energy pose was selected. The docking protocol was assessed by re-docking the co-crystallized ligand within the corresponding binding protein and comparing the generated docking poses with the original co-crystallized poses. 2D and 3D interactions between the ligands and receptors were visualized using Discovery Studio Visualizer v21.1.0.20298.

### Statistical analysis

Statistical analysis was performed by SPSS program version 16. Analysis of variance was performed by one-way ANOVA procedures followed by LSD test. The significance level was set at *P* < 0.05. All results are expressed as the mean ± SD.

## Results

### HPLC–ESI–MS/MS metabolites profiling of *F. lyrata* bark extract

**Fig. 1:**
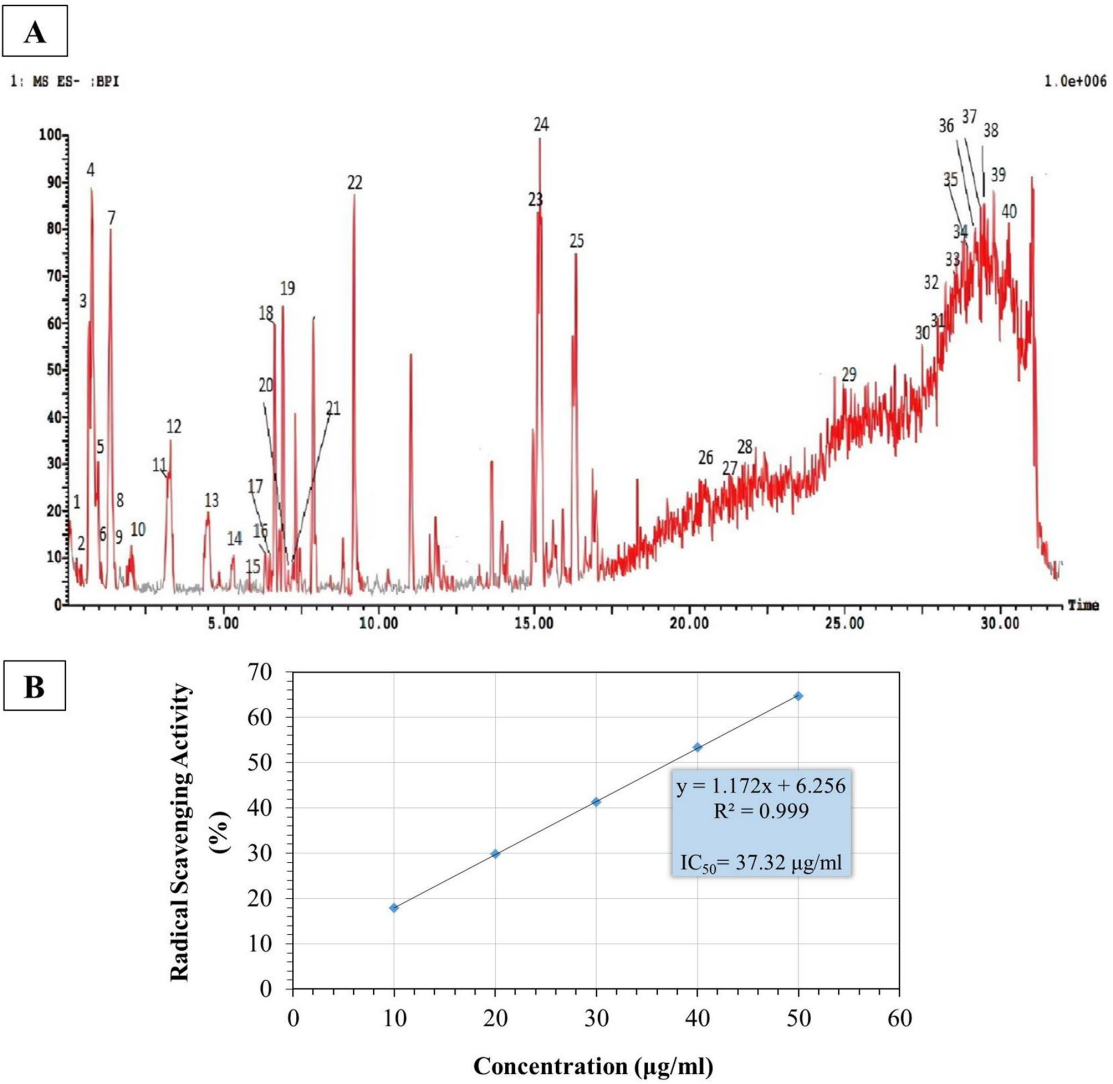
(**A**): HPLC/Ms/Ms chromatogram for *F. lyrata* extract. (**B**): DPPH radical-scavenging activity of the *F. lyrata* extract

HPLC-ESI-MS/MS base peak chromatogram of the extract was shown in (Fig. 1A). We put on view the identification of 40 compounds found in the hydroalcoholic extract of *F. lyrata* bark. The compounds were tentatively detected after comparing their molecular weights, retention times, and mass (MS/MS) fragmentation models with literature. The phytochemical compounds detected in the *F. lyrata* bark extract were sorted into 12 flavonols, six flavones, 15 hydroxycinnamic acid derivatives, and four hydroxybenzoic acids. Flavonols with their glycosides were principally identified as kaempferol, quercetin, and isorhamnetin derivatives. Flavones were identified as luteolin, apigenin, isovitexin, and chrysoeriol, along with their glycosides. Hydroxycinnamic acid derivatives were identified as quinic acid, coumaric acid, ferulic acid, and their derivatives. Hydroxybenzoic acids were identified as protocatechuic acid, 4-hydroxybenzoic acid, vanillic acid, and syringic acid (Table 3).

**Table 3:** Identified compounds of *F. lyrata* warb. Hydroalcoholic bark extract via HPLC/MS/MS

No.	Ret. Time	[M-H]-	Area %	Compound	Fragments
1	0.08	336.8481	0.04	Coumaroyl quinic acid [34]	236, 190, 147
2	0.37	485.8563	0.05	Coumaric acid derivative [35]	308, 236, 208
3	0.68	181.1767	0.70	Dihydro caffeic acid [36]	159
4	0.76	191.1872	1.93	Quinic acid [37]	181
5	0.96	253.1964	0.48	Caffeoyl glyceride [38]	142, 133
6	1.05	447.9796	0.03	Kaempferol-O-Glucoside [39]	285, 243, 218, 161
7	1.36	153.0914	1.74	Protocatechuic acid [39]	153
8	1.50	447.3445	0.04	kaempferol O-galactoside [39]	285, 243,153
9	1.90	479.2806	0.07	Isorhamnetin-O-glucoside [39]	315, 293
10	2.02	137.1187	0.16	4-Hydroxybenzoic acid [40]	137
11	3.18	431.3451	1.03	Isovitexin [39]	281, 179, 121
12	4.37	163.1207	0.58	Coumaric acid [37]	163
13	4.86	167.1601	0.02	Vanillic acid [41]	167
14	5.33	353.0875	0.09	Caffeoyl quinic acid [40]	236, 191
15	5.83	461.2386	0.03	Apigenin-O-Rhamnoside [35]	415, 371, 323, 229, 178
16	6.34	543.3278	0.10	Diferuloyl quininc acid [35]	329, 261, 190
17	6.49	593.4822	0.07	Luteolin 7-rutinoside [42]	390, 285, 241
18	6.80	193.1443	0.17	Ferulic acid [5]	181, 173
19	6.92	381.3343	0.85	Ethyl caffeoyl quinate [40]	763, 381
20	7.08	487.9969	0.03	Caffeic acid-O-hexoside- O-rhamnoside [35]	395, 387, 259,191
21	7.19	577.3055	0.02	Kaempferol-dirhamnoside [38]	509, 409, 312, 243,198, 171
22	9.20	329.4101	1.33	Quercetin-dimethyl-ether [43]	191
23	15.12	417.5255	1.18	Kaempferol-O-arabinoside [5]	309, 295
24	15.19	295.3683	1.58	Caffeoyl malate [34]	295
25	16.34	595.5813	1.77	Quercetin hexoside pentoside [44]	365, 297, 117
26	21.12	655.2822	0.23	Methoxy quercetin- Glucopyranos-gluco-pyranoside [45]	493, 354, 293, 257, 144
27	21.20	367.3179	0.24	Feruloyl quinic acid [46]	268, 257, 191, 118
28	21.35	473.4404	0.27	Caffeic acid hexoside-O-pentoside [35]	443, 367, 270, 118
29	26.29	622.6012	1.59	Rhamnosyl hexosyl methyl quercetin [47]	459, 353, 265, 146
30	27.22	507.4876	0.73	Isorhamnetin-O-rhamnoside [35]	425, 315, 176, 116
31	27.76	490.7792	0.87	Isorhamnetin-O-glucuronide [35]	434, 339, 315, 257, 106
32	27.87	671.3126	1.24	Dicaffeoyl ferulic acid [46]	571, 445, 381, 176, 157
33	28.25	675.4442	1.18	Feruloyl-O- p-coumaroyl-O- caffeoyl shikimic acid [47]	550, 406, 320, 191, 144
34	28.31	324.8372	1.00	Coumaric acid glucoside [37]	201, 157, 144
35	28.60	284.8969	1.89	Luteolin [42]	200, 184, 144
36	28.83	312.8734	2.48	Chrysoeriol-methyl ether [48]	184, 145
37	29.20	577.8765	3.36	Apigenin-O-rutinoside [39]	386, 325, 157, 150
38	29.48	403.4239	1.66	Syringic acid Derivative [35]	265, 145
39	29.60	492.8787	1.26	Isorhamnetin-O-pentoside [35]	433, 315, 219, 142
40	29.88	385.2675	1.61	Sinapic acid-O-glucoside [49]	265, 169, 147

### Radical scavenging activity of *F. lyrata* bark extract

The radical scavenging assay used to evaluate the in vitro antioxidant activity of *F. lyrata* extract, shown in (Fig. 1B) demonstrated strong scavenging activity when compared to the standard reference, Torolox. The IC_50_ value for Trolox was 16.02 µg/mL, while IC_50_ for the *F. lyrata* extract was 37.32 ug/mL with 429.3 mg TE/g extract.

### Safety of *F. lyrata* bark extract

The results of the subacute toxicity study showed that administration of *F. lyrata* extract at different doses up to 5000 mg/kg did not reveal any obvious clinical signs of toxicity or mortality during the experimental period (data not shown). As a result, the LD_50_ is estimated to exceed 5000 mg/kg. Hence, a dosage of 250 mg/kg of the extract was considered safe for usage as a therapeutic dose in this study.

### Effect of *F. lyrata* on anthropometrical measurements in HFD-fed rats

**Fig. 2:**
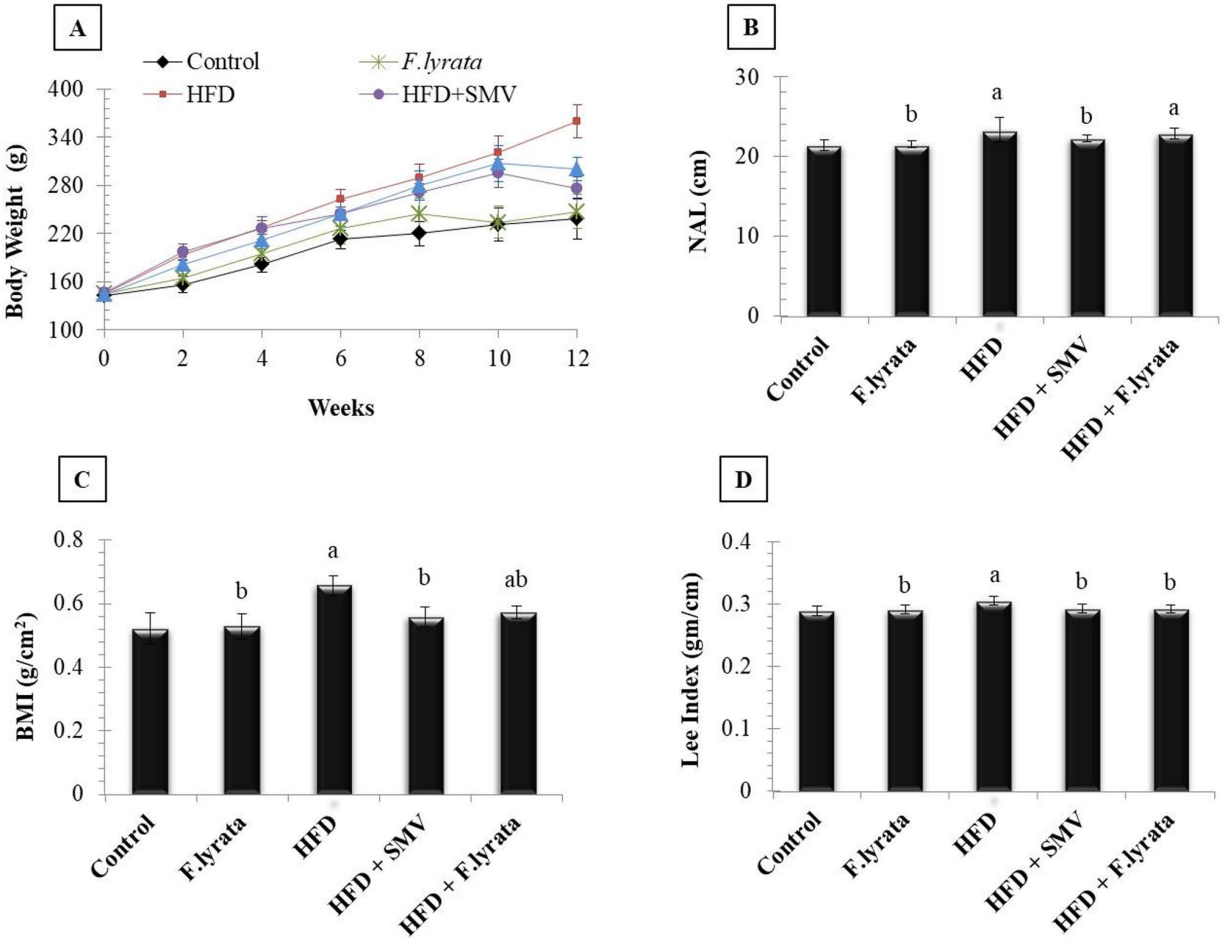
Effect of *F. lyrata* extract on body weight change and anthropometric parameters. **(A)**: Body weight change over the 12-week period, **(B)**: NAL, **(C)**: BMI and **(D)**: Lee index. Data are presented as mean ± SD (*n* = 6). Values are significantly different (^a^*P* < 0.05) compared to control: and values significantly different (^b^*P* < 0.05) in the treatment groups compared to HFD group. BMI = Body weight (g)/length^2^ (cm^2^); Lee index = cubic root of body weight (g) / NAL (cm)

Rats fed HFD showed a significant increase in final body weight, cumulative weight gain, liver index, NAL, BMI, and Lee index compared to control rats. On contrary, *F. lyrata* extract administration to HFD rats significantly reduced these parameters as compared to the HFD group. However, *F. lyrata* extract did not affect the NAL index, (Table 4; Fig. 2).

**Table 4:** The effects of *F. lyrata* extract on body weight, liver weight and liver index in HFD-fed rats

	Initial Weight (g)	Final Weight (g)	%Change from Initial Weight	Liver Weight (g)	Liver Index
Control	143.22 ± 4.46	238.44 ± 35.09	66.02 ± 19.77	7.91 ± 1.46	3.29 ± 0.17
*F.lyrata*	145.40 ± 5.41	247.38 ± 30.01^b^	69.78 ± 15.61 ^b^	9.44 ± 1.25^b^	3.77 ± 0.17^ab^
HFD	145.49 ± 5.79	359.92 ± 51.05^a^	146.69 ± 26.80^a^	15.25 ± 2.11^a^	4.24 ± 0.25^a^
HFD + SMV	146.94 ± 5.69	276.73 ± 14.39^ab^	88.23 ± 13.75^ab^	10.71 ± 0.49^ab^	3.87 ± 0.10^ab^
HFD + *F.lyrata*	144.40 ± 4.58	300.39 ± 14.74^ab^	108.04 ± 13.80^ab^	11.14 ± 1.25^ab^	3.70 ± 0.36^ab^

Data were expressed as the mean ± SD (*n* = 6 in each group). ^a^ *P* < 0.05 vs. Control group; ^b^*P* < 0.05 vs. HFD group.% change in weight = (final weight-initial weight)/initial weight ×100

### Effect of *F. lyrata* extract on liver enzymes and lipid profile in HFD-fed rats

**Fig. 3:**
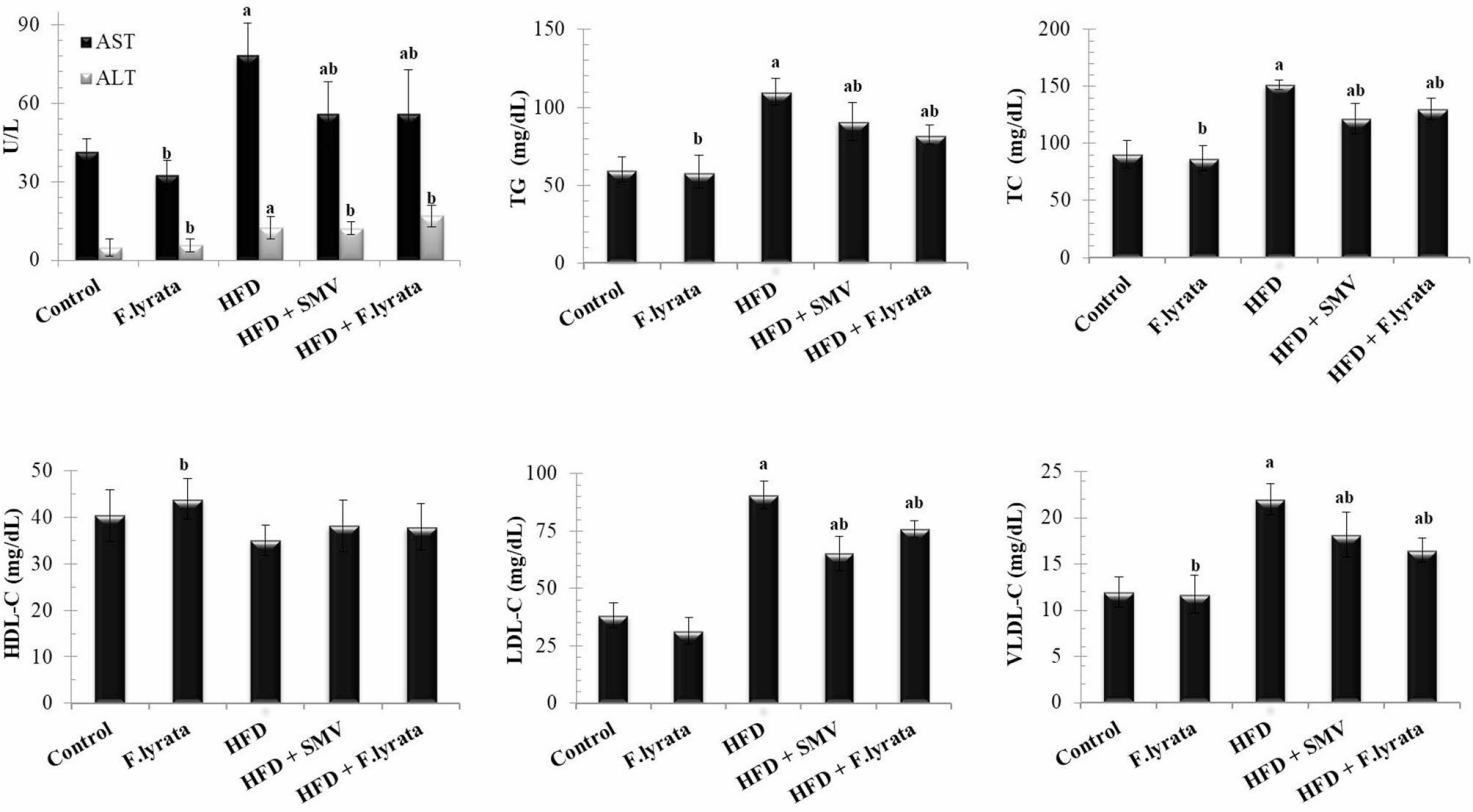
Effect of *F. lyrata* extract on serum aminotransferases and lipid profile. Data are presented as mean ± SD (*n* = 6). Values are significantly different (^a^*P* < 0.05) compared to control: and values significantly different (^b^*P* < 0.05) in the treatment groups compared to HFD group

The HFD group showed a significant elevation in liver enzymes (ALT and AST) as well as elevated the levels of TGs, TC, LDL, and VLDL. On the other hand, the level of HDL significantly decreased in comparing to the control group. However, administration of *F. lyrata* extract to HFD-fed rats significantly improved the increase in liver enzymes and significantly ameliorated dyslipidemia as compared to the HFD group. SMV treatment had comparable results as well, (Fig. 3).

### Effect of *F. lyrata* extract on insulin resistance and adipokine hormones in HFD-fed rats

**Fig. 4:**
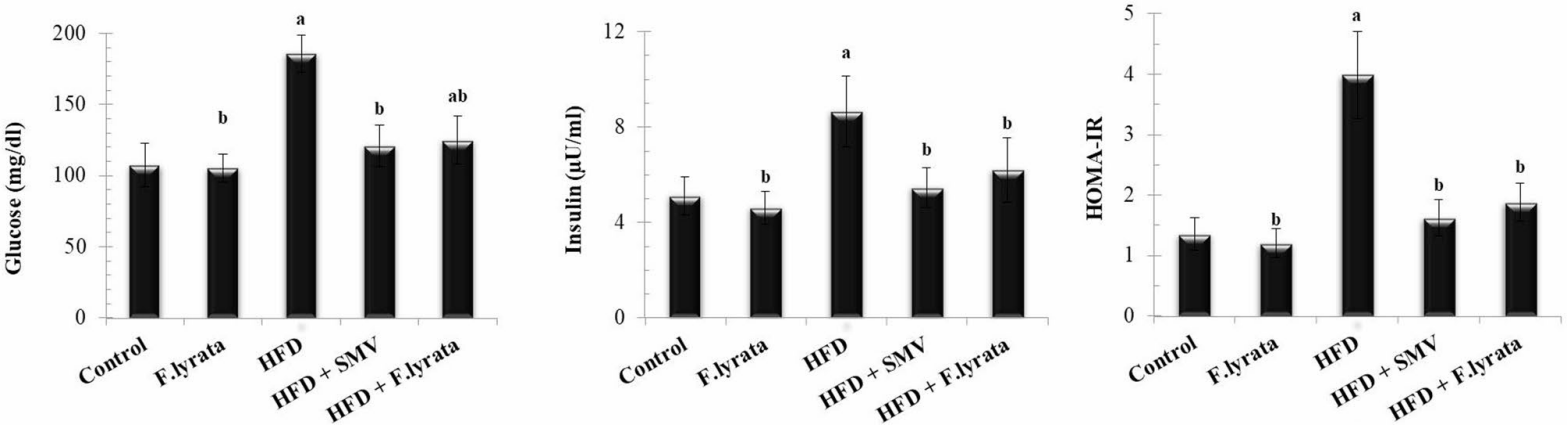
Effect of *F. lyrata* extract on fasting glucose, insulin level and HOMA-IR. Dataare presented as mean ± SD (*n* = 6). Values are significantly different (^a^*P* < 0.05) compared to control: and values significantly different (^b^*P* < 0.05) in the treatment groups compared to HFD group

Figure 4 shows that fasting serum glucose, insulin levels, and HOMA-IR index were significantly elevated in the HFD group compared to control rats, indicating an insulin resistance status. However, treating HFD-fed rats with *F. lyrata* significantly lowered fasting glucose and insulin levels as well as HOMA-IR as compared to the HFD group. 

**Fig. 5:**
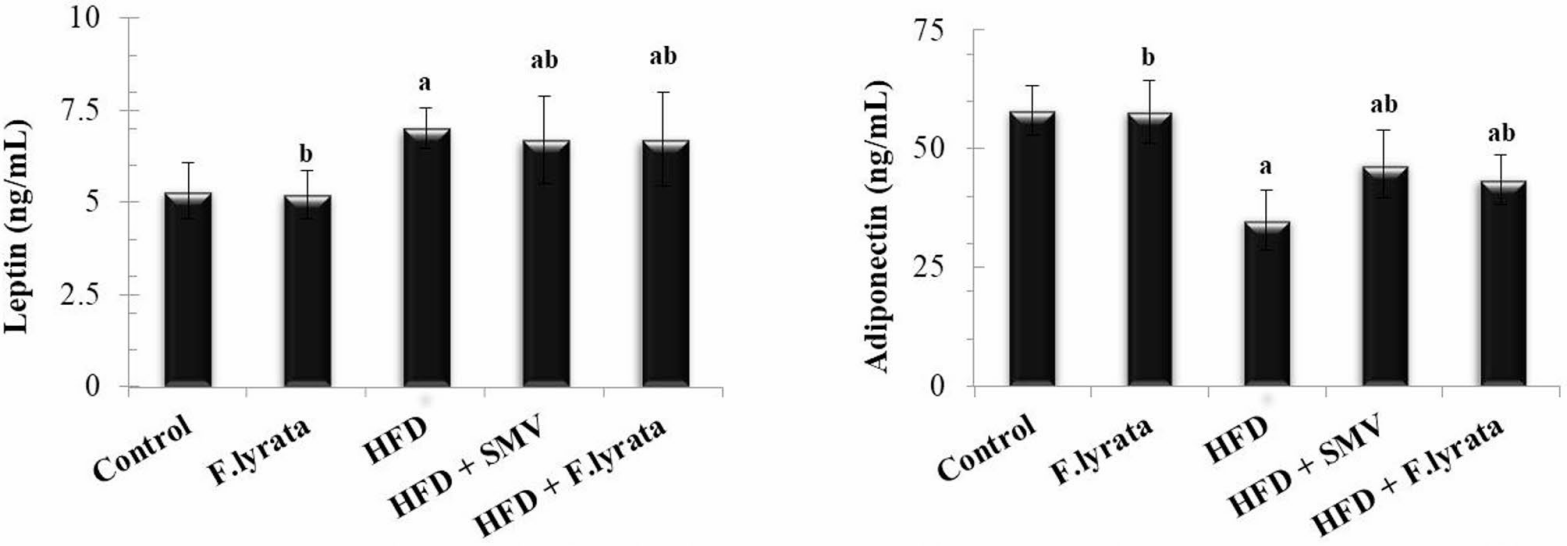
Effect of *F. lyrata* extract on leptin and adiponectin. Data are presented as mean ± SD (*n* = 6). Values are significantly different (^a^*P* < 0.05) compared to control: and values significantly different (^b^*P* < 0.05) in the treatment groups

Moreover, HFD-fed rats exhibited a significant increase in serum levels of pro-inflammatory adipokine (leptin) and a significant decrease in anti-inflammatory adipokine (adiponectin) compared to control animals (Fig. 5). Administration of *F. lyrata* extract ameliorated these changes in serum adipokine levels. Comparable effects were observed after treating the rats with SMV.

### Effect of *F. lyrata* extract on oxidative stress biomarkers in HFD-fed rats

**Fig. 6:**
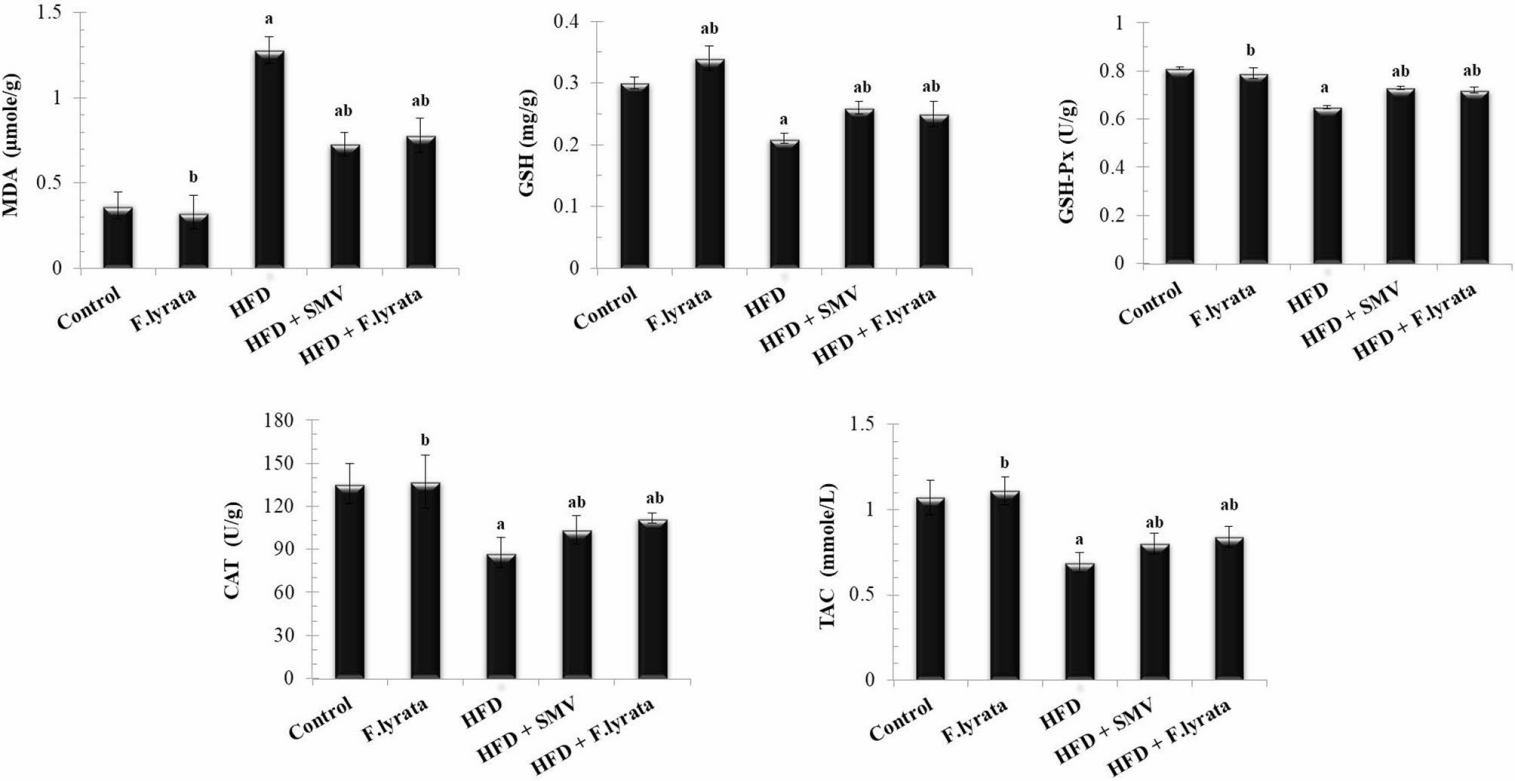
Effect of *F. lyrata* extract on oxidative stress markers. Data are presented as mean ± SD (*n* = 6). Values are significantly different (^a^*P* < 0.05) compared to control: and values significantly different (^b^*P* < 0.05) in the treatment groups compared to HFD group

HFD rats showed a marked increase in MDA and a significant reduction in GSH, GSH-Px, and CAT as compared to control rats. Meanwhile, serum TAC showed significantly decreased levels compared to the control. However, treatment with *F. lyrata* extract significantly reduced these alterations in such biomarkers (Fig. 6).

### Effect of *F. lyrata* extract on expression of hepatic lipogenic genes in HFD-fed rats

**Fig. 7:**
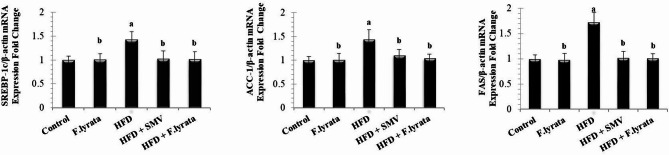
The effect of *F. lyrata* extract on the expression of SREBP-1c, ACC-1 and FAS. Data are presented as mean ± SD. Values are significantly different (^a^*P* < 0.05) compared to control: and values significantly different (^b^*P* < 0.05) in the treatment groups compared to HFD group

Relative expression of three lipogenic genes, SREBP-1c, ACC1, and FAS, is shown in (Fig. 7). Our results revealed a significant increase in the expression of all genes in HFD-fed rats compared with the control group. Treatment with *F. lyrata* extract in the HFD-model rats showed significant downregulation of the studied genes compared with the HFD group. These results were similar to those recorded in SMV group.

### Effect of *F. lyrata* extract on liver morphology and histopathology

**Fig. 8:**
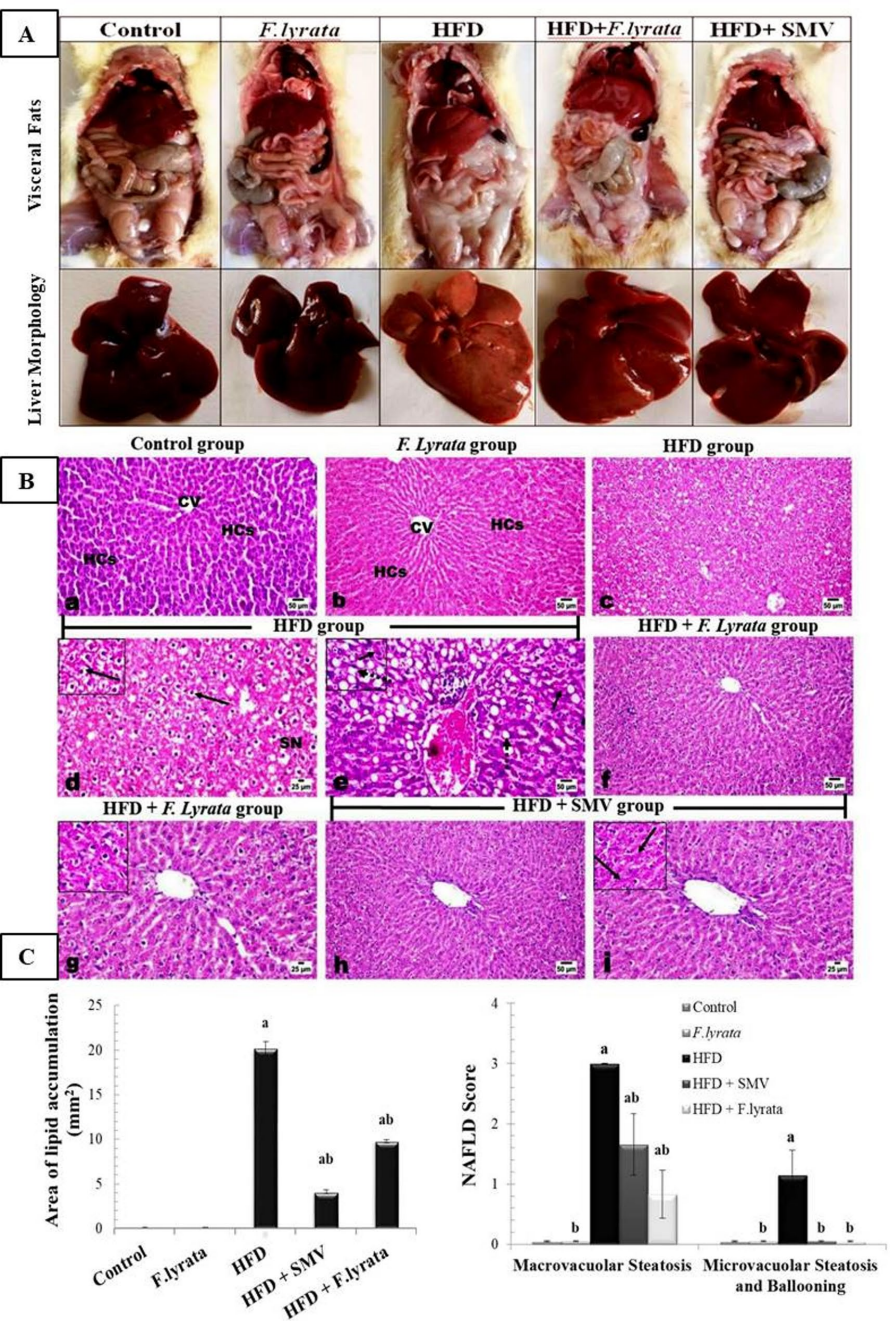
**(A)**: External features of abdominal fats and liver. **(B)**: Photomicrographs of liver sections stained with H&E. Control and *F. lyrata* groups show normal histological structure of central vein (CV) and hepatic cells (HCs). HFD group shows diffuse hepatic steatosis markedly of macrovesicular type, swollen hepatocytes with presence of one or more large fat vacuoles (arrow), signet ring appearance of some cells (dashed arrow), spotty necrosis (SN) that sometimesreplaced by inflammatory cells (circle) and single cell necrosis (short arrow). HFD + *F. lyrata* extract group shows mild degree of hepatocellular vacuolar degeneration (upper insert). HFD + SMV group showing scattered hepatocellular vacuolar degeneration and some single cell necrosis (arrow). **(C)**: Hepatic lipid accumulation area and NAFLD activity score. Data are presented as mean ± SD. Values are significantly different (^a^*P* < 0.05) compared to control: and values significantly different (^b^*P* < 0.05) in the treatment groups compared to HFD group

The morphological features of abdominal fat and liver are shown in (Fig. 8A). HFD-fed rats had increased visceral fat, and the liver lost its normal color and turned pale yellow. *F. lyrata* extract reversed these changes. Liver histopathological observations are shown in (Fig. 8B). The liver sections of normal control as well as the *F. lyrata* group revealed normal histological architecture. In contrast, the liver sections from the HFD group showed variable degrees (moderate to severe) of diffuse hepatic steatosis of the macrovesicular type. Hepatic lipid accumulation was seen as droplets of fats, and hepatocytes were markedly swollen, and large fat vacuoles were found, which in most cases pushed the nucleus to one side, presenting a signet ring shape with activated Kupffer cells and spotty necrosis and single-cell necrosis. The spotty necrotic areas were sometimes replaced by mononuclear inflammatory cells. Zones 2 and 3 were the most affected zones. Microvesicular steatosis was also noticed in a scattered manner. Examination of liver sections of the treated groups revealed marked improvement in the steatosis state of the hepatic cells. *F. lyrata* could efficiently protect the hepatic cells against cellular accumulation of fats; whereas, the hepatic cells showed a moderate degree of vacuolar degeneration with scattered single-cell necrosis cells. Treatment with SMV revealed also retraction of the hepatic steatosis with scattered appearance of hepatocellular vacuolar degeneration and necrosis. Histopathological assessments of the lipid accumulation area and NAFLD score, including steatosis, lobular inflammation, and ballooning of hepatocytes, are shown in (Fig. 8C).

### Effect of *F. lyrata* extract on immunohistochemical analysis of TNF-α and IL-6

**Fig. 9:**
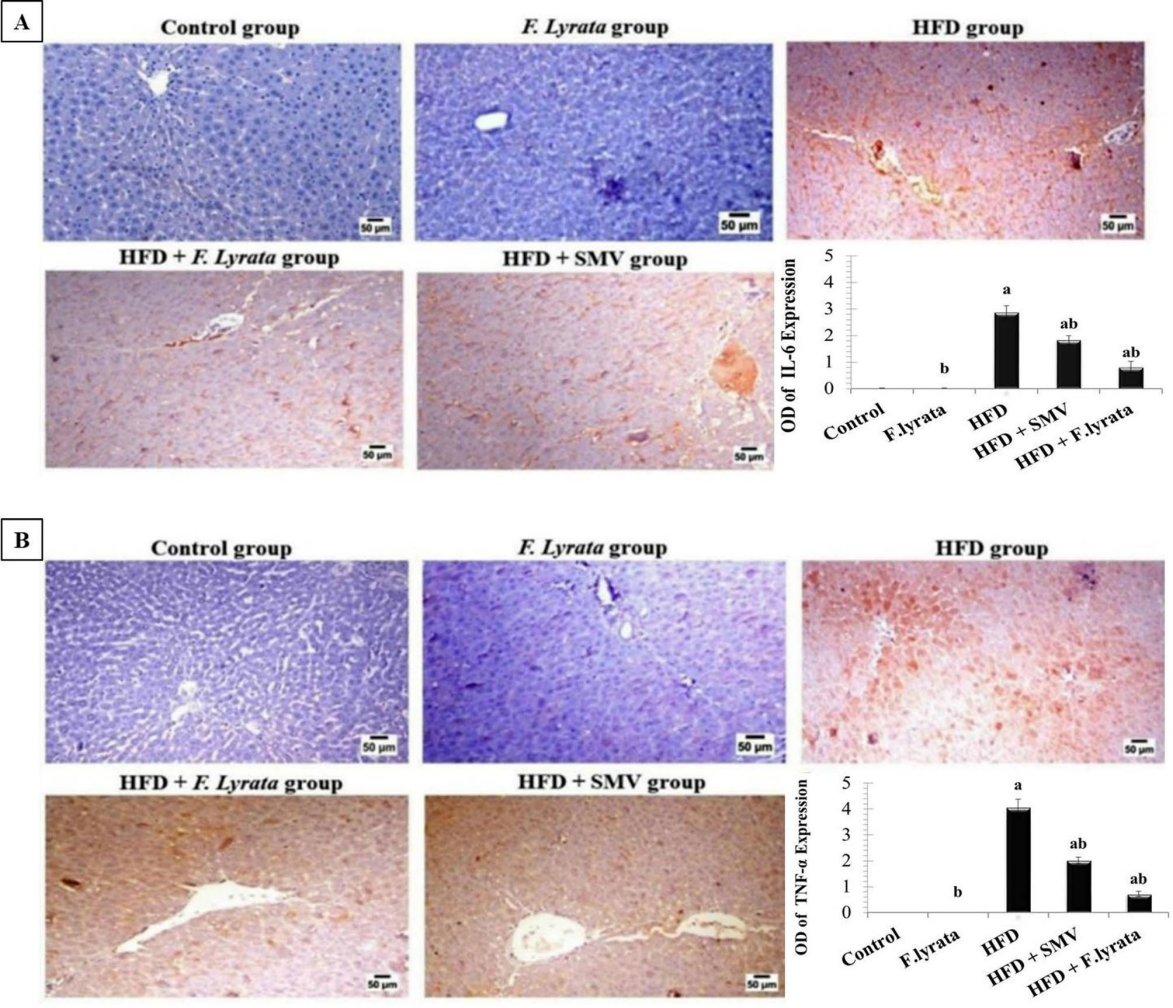
Photomicrograph for immunohistochemical analysis and immune-expressive area percentage. of **(A)**: IL-6 and **(B)**: TNF-α in the liver sections of all experimental groups. Quantification of IL-6 and of TNF-α positive brown colour presented as optical density. Data are expressed as mean ± SD. (*n* = 6) for each group. Values are significantly different (^a^*P* < 0.05) compared to control: and values significantly different (^b^*P* < 0.05) in the treatment groups compared to HFD group

Immunohistochemical expression and quantitative analysis of the immunoexpressed area percentage of IL-6 and TNF-α shown in Fig. 9, revealed that, the liver sections from both the control and *F. lyrata*-treated groups revealed negative immune-expression of IL-6 and TNF-α. In contrast, the livers of HFD rats revealed marked expression of IL-6 and TNF-α compared to the control groups. Decreased immune-expression of both IL-6 and TNF-α was observed in the *F. lyrata*-treated rats, and the SMV-treated group.

### Docking study

Docking simulation of each co-crystallized ligand to its corresponding protein resulted in perfect agreement between the docking poses and the original poses with small RMSD for both proteins (0.763 and 0.382 Å for PPAR-γ and LXR-α, respectively), ensuring the validity of the employed docking protocol.

**Fig. 10:**
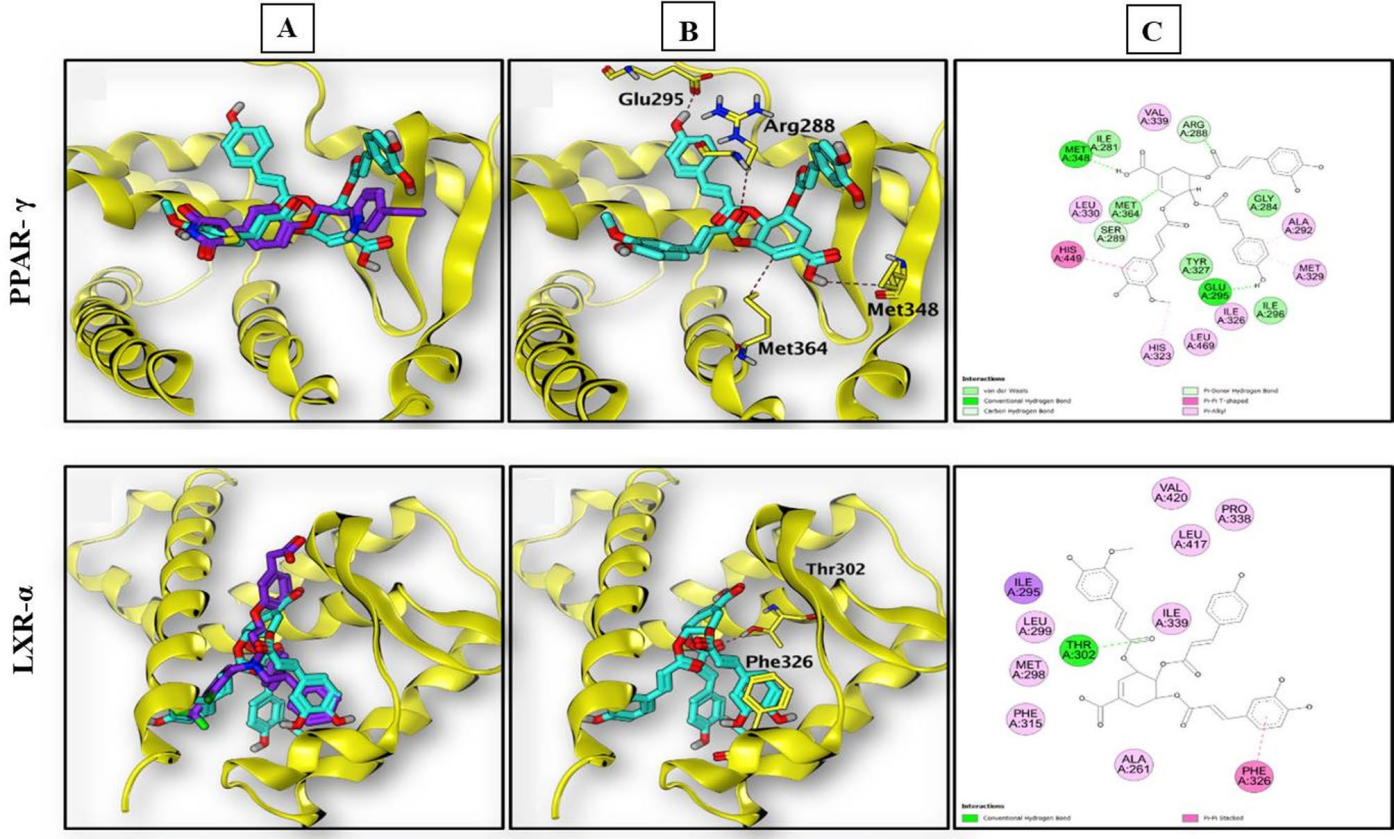
The top scoring pose of feruloyl-O-p-coumaroyl-O-caffeoyl shikimic acid (compound 33) in the ligand binding domain of PPAR-γ and LXR-α. **(A)**: Superposition of compound 33 (aqua) with pioglitazone or GW3965 (magenta) in the active sites of PPAR-γ or LXR-α, respectively. **(B)**: 3D depiction of the binding mode of compound 33 (aqua)displaying the key interactions (dashed line) with pocket residues. **(C)**: 2D presentation of the binding interactions and the active site residues

The binding affinities of the co-crystallized ligands and the top-scoring compounds, as well as their interactions with the binding sites, are compiled in Table 5. Most of the identified compounds in the *F. lyrata* extract demonstrated good affinity toward PPAR-γ and LXR-α ligand-binding domains. However, the best binding affinities were displayed by the flavonoid diglycosides, diferuloyl quinic acid, and caffeic acid diglycosides, with docking scores comparable to or even higher than those achieved by the co-crystallized ligands. Feruloyl-O-p-coumaroyl-O-caffeoyl shikimic acid (33) demonstrated superior binding affinity toward both targets with docking scores of 13.29 and 14.42 for PPAR- γ and LXR-α, respectively. Regarding the interaction of compound (33) with the PPAR-γ binding site, it was found that it can form three hydrogen bonds with the side chains of the essential pocket residues Glu295, Met348, and Met364. Moreover, it interacts with the LXR-α binding site by forming a hydrogen bond with the side chain of Thr302 and is further stabilized in the pocket by hydrophobic interaction with Phe326, (Fig. 10).

**Table 5:** Docking summery of the top scoring identified phytoconstituents in *F.lyrata* extract with the binding sites of PPAR-γ (pdb: 5Y2O) and LXR-α (pdb: 3IPQ)

Compound	PPAR-γ (pdb: 5Y2O)			LXR-α (pdb: 3IPQ)		
	Binding affinity ΔG (kcal/mol)	Molecular interactions (E in kcal/mol)	Amino acid residues	Binding affinity ΔG (kcal/mol)	Molecular interactions (E in kcal/mol)	Amino acid residues
Co-crystalized ligand (RMSD)	-9.824 (0.763)	H-bond (-1.6) H-bond (-3.2) H-bond (-4.1) H-bond (-1.1)	Met 348 Ser 289 His 323 Gln 286	-12.687 (0.382)	H-bond (-0.5) H-bond (-2.2) H-bond (-5.8) H-bond (-1.3) H-bond (-6.5) Ionic (-5.1) Ionic (-4.8)	Phe 257 Arg 305 Arg 305 Leu 316 Leu 316 Arg 305 Arg 305
Diferuloyl quininc acid	-12.01	H-bond (-1.8) H-bond (-0.5) Pi-H (-0.8) Pi-H (-1.3) Pi-H (-0.8)	Cys 285 Met 364 Ser 342 His 449 Leu 469	-11.97	H-bond (-2.6)	Glu 267
Luteolin 7-rutinoside	-12.59	H-bond (-1.0) H-bond (-0.9) H-bond (-2.5) H-bond (-0.6) Pi-H (-0.6) Pi-H (-0.8)	Met 364 His 323 His 449 Gln 286 His 449 His 449	-12.67	H-bond (-4.1) H-bond (-5.1) Pi-Pi	Glu 267 Glu 267 Phe 315
Caffeic acid- O-hexoside-O-rhamnoside	-11.84	H-bond (-1.1) H-bond (-1.2) H-bond (-1.3 H-bond (-1.1) H-bond (-0.5) H-bond (-1.8) Pi-cation (-1.0)	Leu 340 Cys 285 Met 364 Ile 326 Glu 343 His 323 Arg 288	-10.97	H-bond (-6.4) H-bond (-1.3) H-bond (-2.0) H-bond (-0.9) H-bond (-0.9) Pi-H (-0.8)	Glu 267 Phe 257 Met 298 Arg 305 Leu 316 Phe 326
Kaempferol-dirhamnoside	-11.82	H-bond (-0.6) H-bond (-1.0) H-bond (-1.7) H-bond (-1.8) Pi-H (-0.7)	Cys 285 Cys 285 Tyr 473 His 449 Cys 285	-12.41	H-bond (-1.8) H-bond (-0.7) Pi-H (-0.5)	Glu 267 Ser 264 Met 298
Quercetin hexoside pentoside	-11.64	H-bond (-1.0) H-bond (-0.5) H-bond (-1.7)	His 323 Ser 289 Tyr 473	-12.16	H-bond (-0.5) H-bond (-1.1) H-bond (-4.2) H-bond (-0.5) Pi-Pi	Leu 260 Glu 267 Glu 267 Ser 264 Phe 315
Methoxy quercetin- Glucopyranos-gluco-yranoside	-12.68	H-bond (-0.5) H-bond (-1.3)	Glu 343 Lys 265	-12.97	H-bond (-4.4) H-bond (-1.2) H-bond (-2.4)	Glu 267 Met 298 Leu 316
Rhamnosyl hexosyl methyl quercetin	-12.41	H-bond (-0.6) Pi-H (-0.5)	Ser 289 Cys 285	-13.01	H-bond (-0.8) H-bond (-1.4) H-bond (-1.5) Pi-H (-0.5) Pi-Pi Pi-Pi	Glu 267 Glu 267 Glu 267 Thr 302 Phe 315 Phe 315
**Feruloyl-O- p-coumaroyl-O-caffeoyl shikimic acid**	-13.29	H-bond (-1.7) H-bond (-0.8) H-bond (-3.7)	Met 348 Met 364 Glu 295	-14.42	H-bond (-2.2) Pi-Pi	Thr 302 Phe 326
Apigenin-O-rutinoside	-11.8	H-bond (-0.7) H-bond (-1.4) H-bond (-0.7) H-bond (-1.5)	Cys 285 Ser 289 His 449 Tyr 473	-12.54	H-bond (-3.9) H-bond (-0.9) Pi-Pi	Glu 267 Met 298 Phe 315

## Discussion

Non-alcoholic fatty liver disease, recently termed metabolic dysfunction-associated steatotic liver disease (MASLD), is a serious public health issue that is a direct result of the obesity pandemic [50]. Fat accumulation, lipid metabolic disorder, insulin resistance, oxidative stress, and chronic inflammation are the main risk factors for NAFLD development and could be considered potential therapeutic targets for NAFLD treatment [51]. However, there is currently no FDA-approved drug that is reliably effective in treating NAFLD and with the increasing incidence worldwide, there is still a need for novel alternative approaches [52]. Several studies have investigated polyphenols with numerous underlying mechanisms as potential therapeutic agents for treating NAFLD [53]. Consequently, we are interested in identifying the polyphenolic components, particularly flavonoids, of *F. lyrata* bark extract and investigating its possible anti-NAFLD activity in rats.

HPLC–ESI–MS/MS has been considered as an important technique in the identification of phytochemical components, and the MS/MS spectrum is an indicatory fingerprint for every compound in the plant extract, even if they are similar in molecular formula [54]. In the present study, HPLC/MS/MS analysis of *F. lyrata* bark extract identified 40 polyphenolic compounds. Recently, Rufino et al. [55] revealed the significant roles of the polyphenols against NAFLD by targeting different pathways via different mechanisms of action.

In the present study, feeding rats HFD for 12 weeks increased weight gain, BMI, liver weight, and liver index. This could be due to visceral and hepatic fat accumulation; this was confirmed by histological examination of liver sections of the HFD group, which showed macrovesicular steatosis reached more than 66% in some cases. Administration of *F. lyrata* extract significantly suppressed HFD-induced steatosis as shown by the reduction of fat vacuoles, and lipid droplets infiltrating hepatocytes in treated rat livers, thereby preventing lipotoxicity. Moreover, *F. lyrata* reduced liver injury, as observed by significant improvement in serum transaminases, the earliest indicators of hepatic impairment, suggesting a hepatoprotective effect of *F. lyrata*. This protective effect might be attributed to the presence of flavonoids in *F. lyrata* extract, particularly quercetin and vitexin, as well as some phenolic compounds such as quinic acid. These compounds are reported to be promising hepatoprotectants due to their ability to inhibit oxidative stress and hepatocellular apoptosis [55–58].

*F. lyrata* extract has been shown to significantly ameliorate dyslipidemia, which is another hallmark of HFD-induced NAFLD [59]. This could be due to the presence of isorhamnetin, quercetin, apigenin, luteolin, and kaempferol, the major flavonoids in *F. lyrata* bark extract, along with the phenolic compound, ferulic acid. These compounds have been documented to reduce fat accumulation by improving plasma lipid levels, promoting fatty acid omega-oxidation, and modulating lipid metabolism [60–65]. These results suggest a hypolipidemic effect of *F. lyrata*, which might explain the decrease in hepatocyte fatty infiltration and the recovery of the liver histology.

According to Klop et al. [66], the accumulation of lipids in the liver contributes significantly to the development of insulin resistance, which is one of the major risk factors for NAFLD onset and progression from steatosis to non-alcoholic steatohepatitis. Therefore, improving insulin resistance holds the key to mitigate NAFLD progression [67]. In the present study, we observed a significant reduction in insulin resistance by *F. lyrata* treatment in the NAFLD model, as evidenced by hypoinsulinemia, hypoglycemia, and reduced HOMA-IR. Flavonoids in the extract are the main reason behind this effect, through their ability to reduce fat accumulation or modulate insulin signalling pathways in liver tissues [68, 69]. Compounds such as quercetin, apigenin, and chrysoeriol, which have the potential to improve insulin resistance, enhance insulin secretion and reduce blood glucose levels [62, 65, 70].

Ma et al. [63] reported that elevated hepatic lipid accumulation leads to lipotoxicity that elevates reactive oxygen species in the liver, resulting in oxidative stress. Oxidative stress further exacerbates lipid accumulation, impairs the antioxidant status in hepatocytes, causing their damage, and ultimately leads to cell death [71, 72]. Our results revealed that *F. lyrata* effectively reduced hepatic oxidative stress, which most likely occurred by enhancing antioxidants and suppressing lipid peroxidation. This demonstrates the antioxidant activity of *F. lyrata* bark extract, as supported by the results of the DPPH assay. This antioxidant effect is a consequence of the flavonoids and phenolic compounds in the extract.

Oxidative stress and lipotoxicity can induce inflammatory responses [73] by activating the most prevalent inflammatory adipokines, such as leptin, TNF-α, and IL-6, and reducing anti-inflammatory adipokines, as adiponectin, this can lead to chronic inflammation in the hepatocytes [67, 74, 75]. Hyperleptinemia can enhance hepatic fat accumulation and promote inflammation by upregulating IL-6 and TNF-α expression. On the other hand, adiponectin can prevent fat accumulation by enhancing fatty acid oxidation and can suppress inflammation by downregulating hepatic TNF-α expression [66, 73]. Consequently, new therapies that may reduce leptin and enhance adiponectin concentrations could offer anti-inflammatory effects and protect the liver from NAFLD. This is clearly demonstrated in our work, where *F. lyrata* treatment effectively attenuated the inflammatory response induced by HFD by modifying serum levels of leptin and adiponectin, as well as reducing the hepatic TNF and IL-6 expressions. According to Shabalala et al. [67] this is considered one of the quercetin mechanisms to improve NAFLD and hepatic steatosis. Furthermore, chrysoeriol, a major flavone present in the extract, can directly down regulate pro-inflammatory cytokines like IL-6 and TNF-α [70].

Finally, a HFD contributes to NAFLD pathogenesis by stimulating de novo lipogenesis in the liver. De novo lipogenesis is controlled by increasing the expression of SREBP-1c, a master lipogenic transcriptional factor, and its target lipogenic enzymes, ACC-1 and FAS [67]. In the present study, administration of *F. lyrata* reduced the expression of SREBP-1c, FAS, and ACC-1 in the liver tissue. Quercetin, luteolin, and ferulic acid were reported to attenuate lipogenesis and reduce NAFLD severity by suppression of SREBP-1c and FAS expression [60, 63, 76]. Thus, the effect of *F. lyrata* on de novo lipogenesis may mostly be attributed to these compounds. Furthermore, LXR-α and PPAR-γ are important regulators of lipogenesis in the liver. LXR-α has been proved to increase hepatic lipid accumulation and stimulate transcription of the SREBP-1c, FAS, and ACC-1 [77]. PPAR-γ induces the de novo lipogenic enzymes ACC-1 and FAS [78]. Therefore, we performed computational docking analysis of *F. lyrata* polyphenols with LXR-α and PPAR-γ. Molecular interactions showed a high binding affinity for most polyphenolic compounds in the *F. lyrata* bark extract with LXR-α and PPAR-γ, particularly feruloyl-O-p-coumaroyl-O-caffeoyl shikimic acid. The good affinities of these compounds toward LXR-α and PPAR-γ ligand-binding domains may affect their binding to the promoters of their target lipogenic genes, resulting in a decrease in their transcription. This assumption was confirmed by the downregulation of target lipogenic genes. This is consistent with the findings of Kim et al. [79], who reported that shikimic acid attenuated the mRNA and protein expression of SREBP-1c, FAS, and LXR-α in HepG2 cells.

## Conclusion

**Fig. 11:**
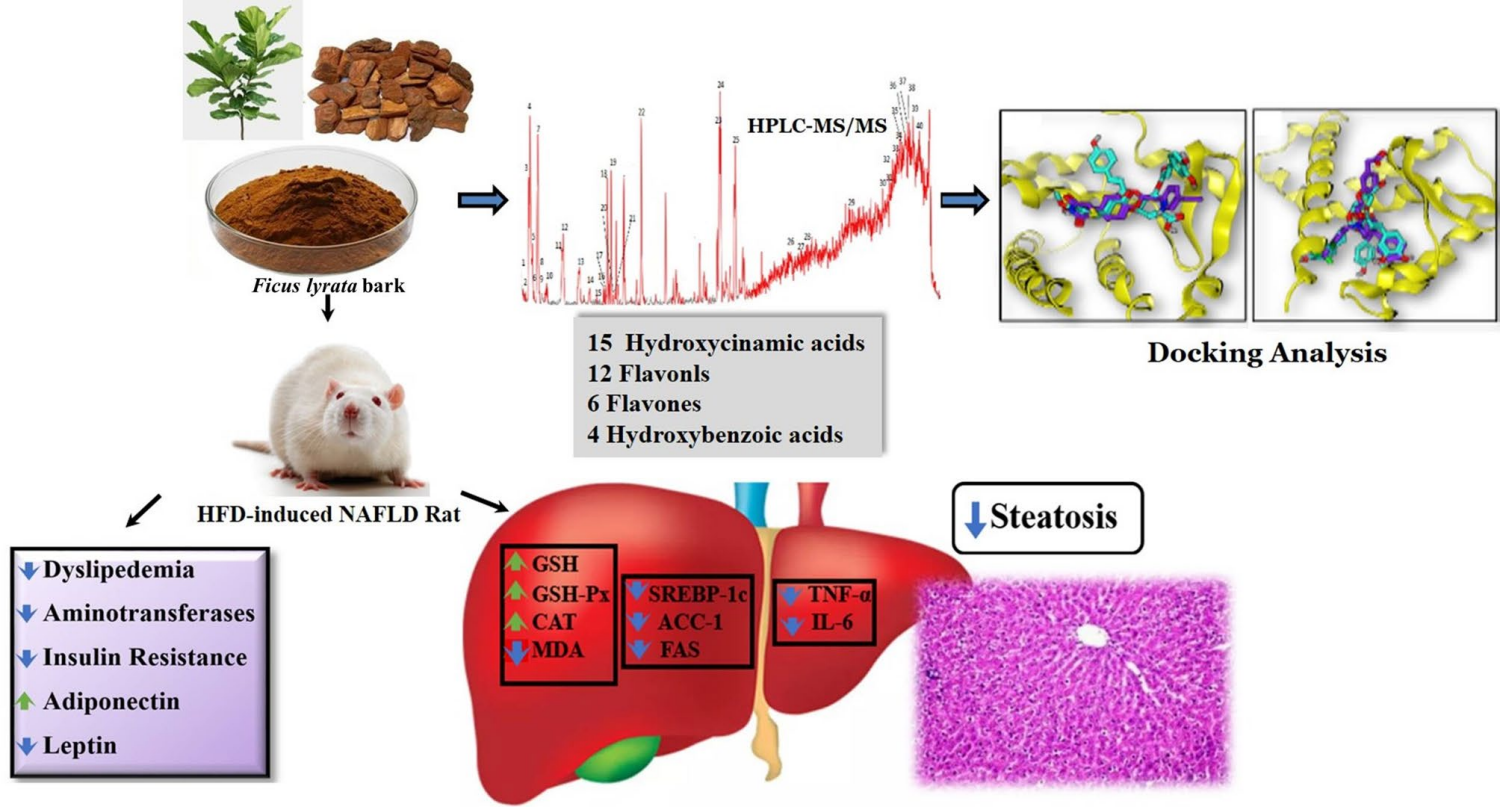
The action of *F. lyrata* on the treatment of HFD-induced NAFLD rats

In conclusion, *F. lyrata* bark extract significantly ameliorated HFD-induced NAFLD in rats. The therapeutic effect of *F. lyrata* against NAFLD, summarized in (Fig. 11), could be attributed to the synergistic effects of various polyphenolic compounds within the extract, which exhibit remarkable capabilities in minimizing de novo lipogenesis, enhancing insulin sensitivity, regulating adipokines, stimulating antioxidant enzymes, and mitigating inflammation. These findings might provide clear evidence supporting the potential therapeutic application of *F. lyrata* extract in the management of NAFLD and its associated metabolic syndrome.

## Data Availability

All data generated or analysed during the current study are available from the corresponding author on reasonable request.

## Abbreviations

ACC-1: Acetyl-Coa carboxylase-1
ALT: Alanine aminotransferase
AST: Aspartate aminotransferase
BMI: Body mass index
CAT: Catalase
DPPH: 2,2-Diphenyl − 1- picrylhydrazyl
ESI: Electrospray ionization
FAS: Fatty acid synthase
GSH: Reduced glutathione
GSH-Px: Glutathione peroxidase
H&E: Hematoxylin and eosin
HDL: High-density lipoprotein
HFD: High fat diet
HPLC: High Performance Liquid Chromatography
HOMA-IR: Homeostasis model assessment of insulin resistance
IL-6: Interleukin-6
LDL: Low-density lipoprotein
LXR-α: Liver X receptorα
MDA: Malondialdehyde
NAFLD: Non-alcoholic fatty liver disease
NAL: Naso-anal length
PPAR-γ: Peroxisome proliferator-activated receptorsγ
SREBP-1c: Sterol regulatory element-binding protein-1c
TAC: Total antioxidant capacity
TC: Total cholesterol
TG: Triglyceride
TNF-α: Tumor necrosis factor-α
VLDL: Very low-density lipoprotein
